# The Chinese version of patient experience with treatment and self-management (PETS vs. 2.0): translation and validation in patients with multimorbidity in primary care in Hong Kong

**DOI:** 10.1186/s41687-024-00765-1

**Published:** 2024-08-02

**Authors:** Zijun Xu, Dexing Zhang, Yang Zhao, Arpita Ghosh, David Peiris, Yiqi Li, Samuel Yeung Shan Wong

**Affiliations:** 1 Jockey Club School of Public Health and Primary Care, The Chinese University of Hong Kong, Hong Kong SAR, China; 2 The George Institute for Global Health, University of New South Wales, Sydney, Australia; 3 The George Institute for Global Health China, Beijing, China; 4 The George Institute for Global Health, New Delhi, Delhi India

**Keywords:** PETS, Treatment burden, Multimorbidity, Primary care, Validation

## Abstract

**Background:**

Validated and comprehensive tools to measure treatment burden are needed for healthcare professionals to understand the treatment burden of patients in China. The study aimed to translate and validate the Chinese version of Patient Experience with Treatment and Self-management (PETS vs. 2.0) in patients with multimorbidity in primary care.

**Methodology:**

The translation process of the 60-item PETS vs. 2.0 followed the Functional Assessment of Chronic Illness Therapy (FACIT) Translation, Formatting, and Testing Guidelines. Computer-assisted assessments were conducted in adult primary care patients with multimorbidity from three general out-patient clinics in Hong Kong. A sample of 502 patients completed the assessments from July to December 2023. Internal reliability was examined using Cronbach’s alphas for each domain of the PETS vs. 2.0. Concurrent validity was assessed through the correlations between different domains of PETS vs. 2.0 with established measures including quality of life, frailty, and depression. Confirmatory Factor Analysis (CFA) with maximum likelihood method was carried out to assess the construct validity.

**Results:**

The mean age of participants was 64.9 years old and 56.2% were female. Internal consistency reliability was acceptable (alpha ≥ 0.70) for most domains. Higher scores of PETS domains were significantly correlated with worse quality of life, higher level of frailty, and more depressive symptoms (*p* < 0.05). In CFA, after setting the covariances on the error variances, the adjusted model revealed an acceptable model fit (χ^2^/df = 1.741; root mean square error of approximation (RMSEA) = 0.038; standardized root mean square residual (SRMR) = 0.058; comparative fit index (CFI) = 0.911; Tucker-Lewis Index (TLI) = 0.903). All standardized factor loadings were 0.30 or above. Significant positive correlations between the latent factors were found for all factor pairs (correlation coefficient < 0.8).

**Conclusions:**

The Chinese version of PETS vs. 2.0 is a reliable and valid tool for assessing the perceived treatment burden in patients with multimorbidity in primary care. All domains and items in the original questionnaires were retained.

## Background

Multimorbidity, defined as the presence of two or more chronic conditions, is a major concern in the primary care setting [1]. The prevalence of multimorbidity in primary care ranged from 12.9 to 95.1% globally [2]. In the Chinese-speaking population, the prevalence was from 25.4 to 54% in Hong Kong and 81.2% in mainland China [3–5]. Due to the complexity of managing multiple chronic conditions simultaneously, multimorbidity poses unique challenges for both patients and healthcare providers. Patients suffering from multimorbidity have multi-level significant treatment burdens. They often face issues such as polypharmacy, high healthcare costs, conflicting treatment recommendations, and the need for coordinated care across different specialities [6, 7]. High treatment burden is an important clinical and primary care issue that may have negative impacts on behavioral, clinical outcomes, and burdens on the healthcare system, such as reduced adherence to treatment, quality of life, increased hospitalization rates and mortality, and increased healthcare cost and health expenditures [8–10]. Multimorbidity services usually involve comprehensive multimorbidity assessment, integrated care planning, and medication reconciliation and deprescribing which distinguishes them from chronic disease management services [11]. It is essential to understand how to deliver multimorbidity services in a way that takes into account patients’ healthcare needs and minimizes treatment burdens [12]. By understanding the patient experience in this population, healthcare professionals can tailor treatment regimens to patients’ realities, enhance adherence, and improve overall patient well-being and outcomes [13–15]. This understanding is essential for developing patient-centered care approaches, optimizing treatment strategies, and fostering effective communication between healthcare providers and patients.

Validated tools such as questionnaires to measure treatment burden are needed for healthcare professionals to understand the treatment burden of patients with multimorbidity. There are some Chinese-validated questionnaires measuring treatment burden available, such as the 10-item Multimorbidity Treatment Burden Questionnaire (C-MTBQ) [8] and the 15-item Burden of Treatment Questionnaire (C-TBQ) [16]. Previous studies suggested that treatment burden is a multi-dimensional construct and several conceptual frameworks of treatment burden were proposed [17, 18]. Although these questionnaires provided brief and structured approaches to assessing the treatment burden, they may still omit several important aspects that patients face, such as impacts of burden (e.g. emotional and social impact) proposed in the frameworks [8, 18]. In order to fully understand how multimorbidity patients experience treatment burden, a comprehensive measurement is needed, which can involve themes such as the things patients must do to maintain good health, the stressors or challenges that exacerbate the feeling of burden, and the impacts of burden [18].

The Patient Experience with Treatment and Self-management Version 2.0 (PETS vs. 2.0) is a valid measure of treatment burden which was originally developed by Eton et al. in 2016 based on the framework [18, 19]. It comprises a total of 60 items, covering 14 domains such as medical information, role functioning, and emotional well-being. PETS vs. 2.0 allows for a comprehensive evaluation of the patient’s experience with treatment and self-management. Furthermore, its adaptability allows for the customization of the questionnaire to suit diverse patient populations or healthcare settings based on specific needs such as stroke survivors [20]. Its breadth and richness also allow for comparisons between different healthcare settings or patient conditions [9]. The PETS vs. 2.0 has been translated and/or validated in many languages in primary care patients with chronic conditions or multimorbidity in recent years, such as Norwegian, Swahili, and Spanish [21–23]. This tool has been widely used in cross-sectional and longitudinal studies in Western countries [24–27]. However, there is no validated Chinese version of this tool so far. The structure of China’s healthcare system, including the role of traditional Chinese medicine alongside Western medicine, may influence how patients experience treatment burden and self-management. Chinese patients may have different attitudes towards healthcare, treatment burden, and self-management compared to Western populations due to cultural differences and differences in health literacy [28]. Over the past decade, medical care in China has gradually transitioned to a primary care-focused approach to reduce the burden arising from chronic conditions [29, 30]. Given the large number of multimorbid patients in primary care in China, researchers and healthcare professionals can better engage with them by making this tool available in Chinese. A validated Chinese version of PETS vs. 2.0 would allow for a more accurate assessment of treatment burden in the Chinese context, effective identification of areas of high treatment burden, better interventions tailored to meet individual patient needs, and evaluation of more dimensions of intervention effectiveness, potentially leading to improved patient care and outcomes. Therefore, this study was conducted to translate PETS vs. 2.0 into Chinese and validate the Chinese version of PETS vs. 2.0 in patients with multimorbidity in primary care. We hypothesized that the Chinese version of PETS vs. 2.0 would have similar psychometric properties and interpretability in treatment burden as the original scale in English-speaking populations.

## Methods

### Study design and participants

The study adopted a cross-sectional design and was conducted in Hong Kong primary care setting from July to December 2023. Subjects with multimorbidity were recruited from three general out-patient clinics (GOPC) in New Territories East in Hong Kong. The inclusion criteria were (1) being 18 years and above, (2) having two or more doctor-diagnosed chronic diseases lasting for at least six months, (3) being able to understand Chinese, and (4) being personally willing to participate after an informed consent process. Ethics was approved by the Joint Chinese University of Hong Kong—New Territories East Cluster Clinical Research Ethics Committee (CREC2023.258). The study was conducted according to the Declaration of Helsinki.

### Instrument

#### Treatment burden

PETS vs. 2.0 is a validated tool measuring treatment burden and self-management [19]. It has 14 content domains with a total of 60 items, including medical information (7 items), medications (7 items), medical appointments (6 items), monitoring health (2 items), interpersonal challenges (4 items), medical & healthcare expenses (5 items), difficulty with healthcare services (7 items), role and social activity limitations (6 items), physical and mental exhaustion (5 items), burdens associated with diet (3 items), exercise/physical therapy (4 items), and use of medical equipment (2 items), as well as bother due to reliance on medicine (1 item) and side effects of medicine (1 item). Screening questions were set for the domains of diet, exercise/physical therapy, and use of medical equipment. These three domains are optional to fill if the participants have no relevant experience. All PETS scores use the same 0 to 100 metric with a higher score indicating more treatment burden. The detailed items in each domain and the scoring calculation methods can be found in Eton et al’s study [19]. The original PETS vs. 2.0 was well-validated in community-dwelling adults suffering from multimorbidity, with Cronbach’s alphas of each domain ranging from 0.80 to 0.94 [19].

#### Multimorbidity checklist

Multimorbidity was defined as having two or more doctor-diagnosed chronic conditions lasting for at least six months. It was assessed using a multimorbidity checklist, which included 17 disease categories (metabolic diseases, cardiovascular diseases, cancer, respiratory diseases, liver and gallbladder diseases, gastrointestinal diseases, musculoskeletal and connective tissue diseases, thyroid diseases, kidney or reproductive system diseases, ear, nose and throat diseases, eye diseases, skin diseases, blood diseases, mental disorders, nervous system diseases, infectious diseases, and congenital diseases) with 72 common chronic diseases. The checklist was made based on chronic conditions listed in previous studies [31, 32], the International Statistical Classification of Diseases 11, and a Delphi consensus study on measuring multimorbidity in research [33].

#### Quality of life

The validated Chinese version of the European Quality of Life Questionnaire the five-level version (EQ-5D-5L) is a standardized tool used to measure the generic health-related quality of life [34]. The EQ-5D-5L contains five questions on mobility, self-care, usual activities, pain/discomfort, and anxiety/depression. EQ-5D-5L comprises five health dimensions and the total score ranges from −1 to 1. Higher scores on the EQ-5D-5L indicate better quality of life.

#### Frailty

Frailty was measured using the Clinical Frailty Scale (CFS) which has been validated in Chinese [35, 36]. The CFS summarizes the overall level of fitness or frailty, which is categorized into 9 levels, ranging from 1 = very fit to 9 = terminally ill. The assessors all passed the CFS Training Module to administer the CFS.

#### Depression

The 9-item Patient Health Questionnaire (PHQ-9) was used to assess depressive symptoms [37]. The Chinese version of PHQ-9 was validated and widely used [37]. Each item is rated on a 4-point scale ranging from 0 (not at all) to 3 (nearly every day). The total score is calculated by adding up the scores of each item and ranges from 0 to 27, with higher scores indicating more depressive symptoms.

### Translation

The whole translation process followed the Functional Assessment of Chronic Illness Therapy (FACIT) Translation, Formatting, and Testing Guidelines (FACIT.org) [38]. The original English version of PETS vs. 2.0 was forward translated into Chinese by two professional bilingual translators, who are fluent in Chinese and English. Subsequently, an independent third native-speaking translator reconciled the two forward translations. The reconciled Chinese version was then back-translated into English by another independent bilingual translator who was blinded to the original questionnaire. The back-translated English version was reviewed by FACITtrans and compared to the original English PETS vs. 2.0 to check if the items were properly translated. Another two independent native speakers, who were public health experts, revised the Chinese version according to practical experience and comments from FACITtrans. The review process was repeated for those problematic items until there was no significant discrepancy between the original version and the translated version.

In the last phase, the translated questionnaire was pre-tested in a convenience sample of 20 primary care patients with multimorbidity from one GOPC in Hong Kong. In the cognitive debriefing, they were invited to give feedback on the general relevance, comprehensibility, and clarity of each translated item, and the difficulty of answering these questions. Comments were reviewed by the research team to finalize the translated PETS vs. 2.0.

### Sample size

Using a criterion of 5–10 participants per question, a sample size of 300–600 was needed [39, 40]. According to Comrey et al’s graded scale, a sample size of 100 corresponds to poor, 200 to fair, 300 to good, 500 to very good, and 1000 to excellent [41]. Therefore, a final sample size of 500 participants was set.

### Procedure

All participants provided written informed consent before they started the interview. The survey was conducted in waiting areas in the three GOPCs by trained research assistants and student helpers in medical-related majors using an online survey platform Qualtrics. The interview platform was developed in Qualtrics. All the participants were voluntarily engaged in the research and could terminate the interview at any time. The survey was anonymous and confidential. Each participant received an incentive of 50 HKD (about 6.4 USD) cash coupon after the interview.

### Data analysis

Continuous variables were summarized as means and standard deviations (SDs), and categorical variables were summarized as numbers and percentages. Concurrent validity was assessed through the correlations between each domain of PETS and quality of life, frailty, and depression. Spearman’s correlation analysis was applied to explore the correlation, with the absolute value of coefficients between 0.50 and 1.00 considered a strong correlation, between 0.30 and 0.50 considered a moderate correlation, and less than 0.30 considered a small or weak correlation [42]. Reliability was examined by assessing internal consistency using Cronbach’s alpha coefficient for each subscale. A Cronbach’s alpha higher than 0.70 was considered as good internal consistency [43].

Confirmatory Factor Analysis (CFA) was carried out to assess the construct validity of PETS vs. 2.0. The dataset was fitted to the measurement model to examine whether the PETS vs. 2.0 conforms to the hypothesized factor structure identified by Lee et al, which contained 12 multi-item domains with 58 items [44]. The two single-item domains were not included in the CFA [44]. The robust maximum likelihood method was used to perform parameter estimation. According to Hu and Bentler’s theory [45], the overall goodness of model fit should be assessed by indices including chi-square statistic to degree of freedom ratio (χ^2^/df), root mean square error of approximation (RMSEA), standardized root mean square residual (SRMR), comparative fit index (CFI) and Tucker-Lewis Index (TLI). The χ^2^/df below 5 or 3, RMSEA below 0.08 or 0.06, SRMS below 0.10 or 0.08, CFI above 0.9 or 0.95, and TLI above 0.9 or 0.95 were considered acceptable or a good model fit, respectively [46]. If the original model fit is unacceptable, the model fit will be improved by adding correlated errors between items within a domain suggested by modification indices [47]. The correlation between latent factors should be less than 0.85, otherwise indicating multicollinearity and problematic discriminant validity [48, 49].

CFA was conducted using MPlus 8 and other statistical analyses were conducted using the Stata version 16.0. The level of significance was set at 0.05 (two-sided) throughout the study.

## Results

### Sociodemographic characteristics

The study reached 1778 primary care patients and 502 participants with multimorbidity who met the inclusion criteria agreed and completed the survey. Their demographic characteristics are presented in Table 1. The mean age was 64.9 years (SD = 10.4), and it ranged from 18 to 90 years. Among the participants, 56.2% were female, 73.1% were married, 70.1% were unemployed including retirement, and 55.8% had a middle school degree. Regarding the number of chronic diseases, 34.3, 28.9, and 36.9% had 2, 3, and 4 or more diseases, respectively.

**Table 1:** Sociodemographic characteristics of the multimorbidity participants in primary care (n = 502)

Characteristics	n	%
**Age**		
<60	106	21.1
60–69	221	44.0
70+	175	34.9
**Gender**		
Male	220	43.8
Female	282	56.2
**Marriage**		
Married	367	73.1
Unmarried	135	26.9
**Employment**		
Employed	150	29.9
Unemployed	352	70.1
**Education**		
Primary school or below	140	27.9
Middle school	280	55.8
Preparatory or above	82	16.3
**Number of chronic diseases**		
2	172	34.3
3	145	28.9
≥4	185	36.9

### Concurrent validity

Concurrent validity was analyzed by calculating the Spearman’s correlations between the 14 domains of the Chinese version of PETS vs. 2.0 and the scores of EQ-5D, CFS, and PHQ-9 (Table 2). All domains were significantly correlated with the scores of the EQ-5D, CFS, and PHQ-9 (*p* < 0.05), with the Spearman’s correlations ranging from −0.18 to −0.47 for EQ-5D, and from 0.12 to 0.25 for CFS, and from 0.24 to 0.68 for PHQ-9.

**Table 2:** The Spearman’s correlation of the domains of the PETS vs. 2.0 with quality of life, frailty, and depression

Domains of PETS vs. 2.0	n^#^	EQ-5D	CFS	PHQ-9
		rho	rho	rho
Medical information	501	−0.32***	0.20***	0.29***
Medications	493	−0.22***	0.17***	0.24***
Medication reliance bother	494	−0.30***	0.12**	0.35***
Medication side effects bother	494	−0.31***	0.22***	0.32***
Medical appointments	502	−0.27***	0.17***	0.31***
Monitoring health	491	−0.23***	0.18***	0.29***
Diet	330	−0.18**	0.12*	0.25***
Exercise or physical therapy	345	−0.22***	0.23***	0.33***
Medical equipment	388	−0.25***	0.18***	0.27***
Relationships with others	502	−0.36***	0.19***	0.45***
Medical and healthcare expenses	487	−0.33***	0.13**	0.36***
Difficulty with healthcare services	486	−0.35***	0.21***	0.34***
Role and social activity limitations	502	−0.38***	0.23***	0.41***
Physical and mental exhaustion	502	−0.47***	0.25***	0.68***

*p < 0.05, **p < 0.01, ***p < 0.001

^#^Number of participants with available data

*CFS* clinical frailty scale, *EQ-5D* European quality of life questionnaire, *PETS* patient experience with treatment and self-management, *PHQ-9* 9-item patient health questionnaire

### Reliability of the psychometric scales

The Cronbach’s alpha coefficients of the subscales of the PETS vs. 2.0 ranged from 0.67 to 0.93 (Table 3). All the coefficients were no less than 0.7, except for diet (alpha = 0.67).

**Table 3:** The Cronbach’s alpha of each domain and the standardized factor loadings of the Chinese version of PETS vs. 2.0

Domain	Cronbach’s alpha (internal reliability)	Item	Factor loadings (construct validity)	
			Original	Adjusted*
Medical information	0.86	MINF1	0.72	0.72
		MINF2	0.67	0.66
		MINF3	0.59	0.54
		MINF4	0.76	0.76
		MINF5	0.71	0.72
		MINF6	0.63	0.60
		MINF7	0.76	0.76
Medications	0.93	MED1	0.80	0.82
		MED2	0.83	0.85
		MED3	0.84	0.85
		MED4	0.80	0.80
		MED5	0.81	0.81
		MED6	0.88	0.86
		MED7	0.80	0.77
Medical appointments	0.89	MAP1	0.75	0.75
		MAP2	0.77	0.77
		MAP3	0.80	0.80
		MAP4	0.75	0.75
		MAP5	0.77	0.77
		MAP6	0.75	0.75
Monitoring health	0.76	MH1	0.77	0.77
		MH2	0.82	0.82
Diet	0.67	DIET1	0.49	0.49
		DIET2	0.73	0.73
		DIET3	0.74	0.74
Exercise/physical therapy	0.76	PT1	0.79	0.79
		PT2	0.83	0.83
		PT3	0.74	0.74
		PT4	0.39	0.39
Medical equipment	0.70	MEQ1	0.90	0.90
		MEQ2	0.61	0.61
Relationships with others	0.83	RLO1	0.72	0.72
		RLO2	0.70	0.70
		RLO3	0.83	0.83
		RLO4	0.76	0.76
Medical expenses	0.89	MEXP1	0.76	0.76
		MEXP2	0.74	0.74
		MEXP3	0.94	0.94
		MEXP4	0.94	0.94
		MEXP5	0.44	0.44
Difficulty with healthcare services	0.70	HCS1	0.58	0.61
		HCS2	0.38	0.41
		HCS3	0.30	0.30
		HCS4	0.70	0.57
		HCS5	0.73	0.60
		HCS6	0.55	0.58
		HCS7	0.40	0.43
Role/social activity limitations	0.92	RAL1	0.74	0.74
		RAL2	0.80	0.81
		RAL3	0.88	0.88
		RAL4	0.89	0.89
		RAL5	0.83	0.83
		RAL6	0.74	0.74
Physical/mental exhaustion	0.89	PMF1	0.76	0.76
		PMF2	0.83	0.83
		PMF3	0.82	0.82
		PMF4	0.71	0.71
		PMF5	0.87	0.87

*After adding correlated errors between items within a domain suggested by modification indices

### Confirmatory factor analysis

All the domains were finished by more than 95% of the participants, except for the domain of diet (n = 330, 65.7%), exercise (n = 345, 68.7%), and medical equipment (n = 388, 77.3%) which were optional based on the own situation of the participants. All the participants had data on at least 8 of the 12 domains. Therefore, confirmatory factor analysis was done on all participants.

**Fig. 1:**
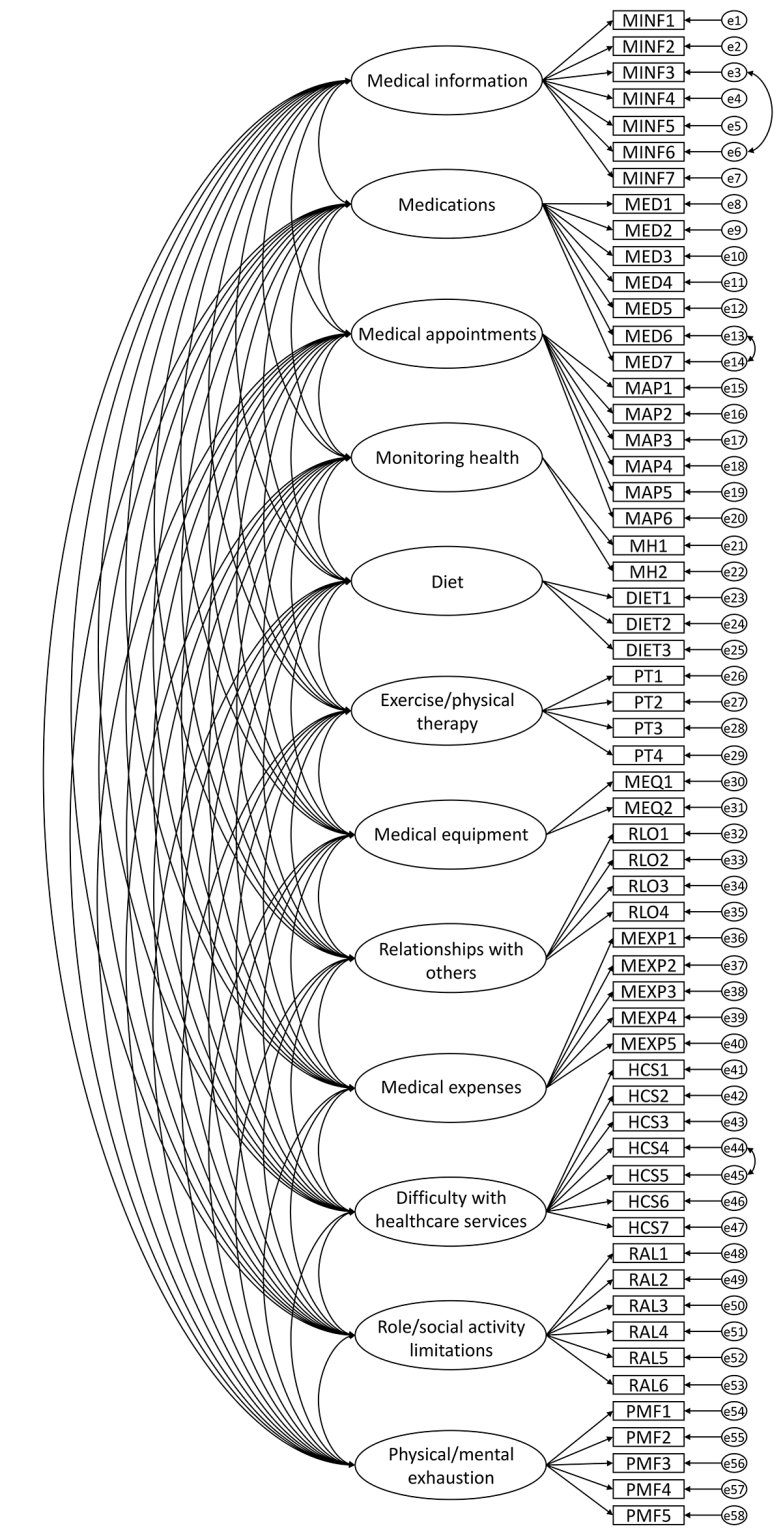
Confirmatory factor analysis showing the 12-factor structure of the Chinese version of PETS vs. 2.0 among primary care multimorbidity patients

The original fit indices revealed a moderate fit approaching acceptable (χ^2^/df = 2.241; RMSEA = 0.041; SRMR = 0.061; CFI = 0.898; TLI = 0.890). To enhance the psychometric qualities of the scale, as suggested by the high modification indices, the original model was modified by assuming correlated measurement errors of HCS4 and HCS5, MINF3 and MINF6, and MED6 and MED7 (Fig. 1). These modifications improved the model fit to be acceptable (χ^2^/df = 1.741; RMSEA = 0.038; SRMR = 0.058; CFI = 0.911; TLI = 0.903). The adjusted model is shown in Fig. 1. All standardized factor loadings were 0.30 or above after adjustment (ranging from 0.30 to 0.94). Factor loadings in the original and the adjusted model are shown in Table 3. Significant positive correlations between the latent factors were found for all factor pairs, with the correlation coefficient ranging from 0.17 to 0.78 (Table 4).

**Table 4:** Factor correlations of the 12-factor model

	MINF	MED	MAP	MH	DIET	PT	MEQ	RLO	MEXP	HCS	RAL	PMF
MINF	1.00											
MED	0.60	1.00										
MAP	0.60	0.78	1.00									
MH	0.69	0.68	0.69	1.00								
DIET	0.35	0.28	0.32	0.41	1.00							
PT	0.24	0.22	0.25	0.48	0.65	1.00						
MEQ	0.49	0.68	0.63	0.71	0.22	0.17	1.00					
RLO	0.41	0.44	0.51	0.46	0.42	0.37	0.31	1.00				
MEXP	0.44	0.35	0.38	0.44	0.33	0.29	0.37	0.30	1.00			
HCS	0.55	0.45	0.64	0.55	0.51	0.38	0.46	0.47	0.56	1.00		
RAL	0.37	0.22	0.33	0.41	0.38	0.35	0.22	0.57	0.35	0.50	1.00	
PMF	0.41	0.30	0.38	0.47	0.42	0.33	0.25	0.58	0.41	0.50	0.65	1.00

*DIET* diet, *HCS* difficulty with healthcare services, *MAP* medical appointments, *MED* medications, *MEQ* medical equipment, *MEXP* medical expenses, *MH* monitoring health, *MINF* medical information, *PMF* physical/mental exhaustion, *PT* exercise/physical therapy, *RAL* role/social activity limitations, *RLO* relationships with others

## Discussion

This study aimed to translate, for the first time, and validate the Chinese version of PETS vs. 2.0 in primary care patients with multimorbidity in Hong Kong. Following the standard translation process of the FACIT guidelines, successful translations and transcultural adaptations were achieved. The results demonstrated acceptable reliability and validity of the translated PETS vs. 2.0 in patients with multimorbidity in the Chinese-speaking subjects, with all 14 domains and 60 items being retained.

Regarding the internal consistency reliability, Cronbach’s alpha coefficients of 11 domains were 0.7 or above, indicating good internal consistency. The reliability of the diet domain (alpha = 0.67) showed moderate internal consistency approaching acceptable. The potential explanations include the small sample size, limited items, item heterogeneity, and sample characteristics. One possible interpretation is that the diet domain was optional and some participants did not get recommendations for healthy eating from healthcare professionals. Therefore, the target sample size of 500 was not reached for this domain. Furthermore, there are only three items in this section, and participants may give quite different answers for the first two items, e.g. agree with “I have to give up too many foods that I like” and disagree with “It is hard to find healthy foods”, which may be due to the different food supply in the local context of Hong Kong from the West. The internal consistency reliability of the Chinese version of PETS vs. 2.0 is comparable to that of the Spanish version (alpha range: 0.62 to 0.92) [23] but is a little bit lower compared to the original English version (alpha range: 0.80 to 0.94) [44]. It may be due to the differences in the patient characteristics and healthcare systems.

Studies have shown that treatment burden was inversely associated with quality of life, physical health, and mental health [13, 50–52]. In the concurrent validity analysis, the data showed significantly small-to-moderate correlations of the Chinese version of PETS vs. 2.0 with quality of life, frailty, and depression. It indicates acceptable concurrent validity of the Chinese version of PETS vs. 2.0 to reflect the quality of life and physical and mental health of multimorbidity patients. The correlations further imply that the challenges and demands of medical treatments can have a significant association with the overall physical and mental well-being of patients. This highlights the importance of considering the treatment burden when addressing the care and support needs of patients to optimize their well-being.

The CFA was conducted to assess the goodness of fit statistics of the 12-domain model. The original CFI (0.898) and TLI (0.890) indices of the model fit were less than 0.900, indicating a moderate fit approaching acceptable. This may be because some items are highly correlated with each other. For example, when assuming correlated measurement errors of certain items within the domain of medical information (i.e., MINF3 and MINF6), medications, and difficulty with healthcare services, the CFI and TLI would reach 0.9. The χ^2^/df, RMSEA, and SRMR in both the original and the adjusted model could reach a good level of model fit. Although all the standardized factor loadings loaded 0.30 or above and met the minimum acceptable standard, some factor loadings were not high for some items. All the factors were kept to be consistent with the original model.

Despite some slight differences between the Chinese and English languages and wording, there was little difficulty in translating the PETS vs 2.0 into Chinese. By having a validated Chinese version of PETS vs. 2.0, healthcare providers can better understand the high treatment burden domains and the factors associated with them in primary care in China. This can help healthcare providers assess treatment preferences, and improve communication quality and patient-centered care, therefore, enhancing treatment adherence and satisfaction in patients. It is also important to assess the validity and reliability of the Chinese version of PETS vs. 2.0 and understand the treatment burden across a broad of general and clinical populations in China. On the basis of these understandings, tailored interventions targeting different domains of treatment burden can be developed. The tool may also be used to evaluate intervention effectiveness in patients. With the increasing adoption of PETS vs. 2.0 around the world, it allows international comparison of treatment burden in different populations.

### Strengths and limitations

The strengths of this study are the translation of a well-established instrument, the PETS vs. 2.0, into Chinese following a standard procedure. This study validated the scale in primary care patients with multimorbidity in Hong Kong, a vulnerable population that experiences a significant treatment burden. This study also has some limitations. First, the convenience sampling method with a low response rate resulted in patients participating in the survey being more likely to be engaged, which will affect the representativeness of primary care patients with multimorbidity in Hong Kong. Second, the chronic diseases were self-reported, although the medical records of some participants could have been double-checked if they had been willing to provide their GOPC numbers to the assessors. There might exist recall bias. Third, other types of reliability were not measured in this study such as test-retest reliability due to difficulties in contacting the participants again after the initial survey in the primary care clinic in Hong Kong within a short time. However, the original validation study showed that the intraclass correlation coefficients of ten domains were ≥0.70, indicating adequate test-retest reliability [44].

## Conclusions

In summary, this study translated the PETS vs. 2.0 into Chinese and undertook reliability and validity tests in Chinese primary care patients with multimorbidity. The Chinese version of PETS vs. 2.0 was found to be a reliable and valid tool for assessing perceived treatment burden in patients with multimorbidity in primary care. All domains and items in the original questionnaires were retained. It can be used by healthcare professionals and researchers with a health background to evaluate patient treatment burden. Future larger-scale studies can be conducted in both Mainland China and Hong Kong to measure the treatment burden of patients.

## Data Availability

The datasets used and analysed during the current study are available from the corresponding author upon reasonable request. The Patient Experience with Treatment and Self-management version 2.0 (PETS vs. 2.0), including all modified, translated, and adapted versions of it, are protected by copyright, © 2016, 2020 Mayo Foundation for Medical Education and Research. All rights reserved. All requests to use copies of this questionnaire should be addressed to Kathleen Yost, PhD yost.kathleen@mayo.edu.

## Abbreviations

CFA: Confirmatory factor analysis
CFI: Comparative fit index
CFS: Clinical frailty scale
C-MTBQ: Chinese version of multimorbidity treatment burden questionnaire
C-TBQ: Chinese version of burden of treatment questionnaire
EQ-5D-5L: European quality of life questionnaire the five-level version
GOPC: General out-patient clinic
HCS: Difficulty with healthcare services
FACIT: Functional assessment of chronic illness therapy
MAP: Medical appointments
MED: Medications
MEQ: Medical equipment
MEXP: Medical expenses
MH: Monitoring health
MINF: Medical information
PETS vs. 2.0: Patient experience with treatment and self-management version 2.0
PHQ-9: 9-item patient health questionnaire
PMF: Physical/mental exhaustion
PT: Exercise/physical therapy
RAL: Role/social activity limitations
RLO: Relationships with others
RMSEA: Root mean square error of approximation
SD: Standard deviation
SRMR: Standardized root mean square residual
TLI: Tucker-Lewis index
χ^2^/df: Chi-square statistic to degree of freedom ratio
